# Observations on Simple Fractures Where the Union Fails

**Published:** 1801-09

**Authors:** A. Carlisle

**Affiliations:** Surgeon to the Westminster Hospital, &c. &c.


### *Observations on Simple Fractures where the Union fails. By* A. Carlisle, Efq. Surgeon to the Weftminfter Hofpital, &c. &c.

---

To Dr. BATTY.

Dear Sir,

One of the unpleafant accidents which occafionally happen in the treatment of fimple Fractures of the Cylindrical Bones, is the failure of union. It may poffibly have occurred to fome of your readers to note particular circumftances either in the general conftitution, the mode of treatment in fuch cafes, or peculiarities in the manner of the fracture. As I have not met with any fatisfactory reafons for the defect of offific union in thofe occafional inftances, I beg leave to ftate the following hiftories, which may perhaps induce gentlemen of greater experience, and better knowledge, to clear up this obfcurity. The following three cafes were the fubjects of memorandums at the time they prefented themfelves, and which record is as follows.

A failor, about forty-five years of age, on board one of His Majefty's fhips of the line on the Jamaica ftation, had his thigh fractured by a fall, and foon afterwards returned home with a fleet. After fifteen months the broken thigh had not acquired any firmnefs, but it was eafily moved, as if a joint was formed at the fracture. The ends of the broken bone had paffed each other two inches. The man was athletic, of dark complexion, chewed much tobacco, and drank fpirituous liquors whenever he could obtain them. By his own account, the treatment of his limb, both as to bandages and reft, had been according to the common routine. This man was induced to undergo a painful operation; both ends of the fractured femur were fawed off to the extent of an inch and a half from each, and although the limb was carefully attended to, yet the union did not obtain, and he remained to the time of his death with a flexible joint in the middle of his thigh.

A young athletic man, between twenty and thirty years of age, by trade a houfe carpenter, had a fimple fracture of the os brachii about its middle; the bandages and fplints in common ufe were applied, and it was concluded as a matter of courfe that his limb would become firm in the ordinary time. At the end of fix months I obferved him ftill ufing the fling, and carrying his fore-arm and hand like a dead limb. On removing the bandages the fracture had not united, and motions of the limb did not occafion pain in the part. This man was a patient in the Weftminfter Hofpital, and had been enjoined low diet and extenfive evacuations, which over anxiety on his part had carried to great extent. His limb had always hung low in the fling, the fore-arm and hand were conftantly œdematous, and the upper arm had ftretched full two inches in length, fo that the ends of the fracture were feparated to this diftance. I faw him twelve months afterwards with a difunited bracheum, having tried a variety of methods to excite union, but all unfuccefsfully.

A foldier, between thirty and forty years old, had his tibia broken and fibula diflocated; he had been treated in a military hofpital, and the lowering plan carried to its full extent. After ten months the broken bone was loofe, although a piece of fplinter appeared to be intervening; it is very probable that this man's limb became firmer, but I have never heard of his fate.

Although cafes of defective offification after fimple fractures are are uncommon, yet when they do happen it is very diftreffing to both the patient and practitioner. Nor is there any decided inference to be drawn from thefe cafes, as to the certain caufe of fuch misfortunes.

The failor's thigh did not fail, becaufe the broken ends were pufhed beyond each other; for this commonly happens to the fame extent, and yet the fracture unites; and the refult of his operation fhewed fomething like a conftitutional deficiency in the offific procefs.

The carpenter's arm muft have been in a favourable pofition for union during the earlier period, for it was kept remarkably ftraight.

The foldier had experienced no other treatment or fymptom beyond what may be confidered as common routine.

In all thefe men the vafcular fyftem appeared fluggifh, their pulfes were flow, and the characteriftics of inflammatory dif-pofition were wanting. Perhaps, in fuch conftitutions where the offific union has once failed, or where the fluggifh, inactive difpofition of the vafcular fyftem is well marked, it may be prudent to watch the natural progrefs of inflammatory fymptoms; to moderate them when actually prefent, *rather than to anticipate their appearance*; and after the thirtieth day, to commence a more generous regimen.

It has not occurred to me to make obfervations on a number of fimilar cafes which I have feen, and which, perhaps, other practitioners may have noticed, with a view to an explanation of this phenomenon. I am,

Dear Sir,

Yours, &c.

*Soho Square, Aug.* 26, 1801. A. CARLISLE.

